# Pulmonary artery intimal sarcoma: poor ^18^F-fluorodeoxyglucose uptake in positron emission computed tomography

**DOI:** 10.1186/1749-8090-8-40

**Published:** 2013-03-07

**Authors:** Dong-Hyup Lee, Tae-Eun Jung, Jang-Hoon Lee, Dong-Gu Shin, Won-Jong Park, Jun-Hyuk Choi

**Affiliations:** 1 Department of Thoracic and Cardiovascular Surgery, College of Medicine, Yeungnam University, Daemyeong 5-dong, Nam-gu, Daegu, South Korea; 2 Division of Cardiology, College of Medicine, Yeungnam University, Daegu, South Korea; 3 Department of pathology, College of Medicine, Yeungnam University, Daegu, South Korea

**Keywords:** Intimal sarcoma, Pulmonary artery, Thromboembolism

## Abstract

Intimal sarcoma of the pulmonary artery is a rare malignant tumor that may be misdiagnosed as chronic pulmonary thromboembolism, even if various imaging techniques are used. We report a case of a 58-year-old man with pulmonary artery intimal sarcoma.^18^F-fleuorodeoxyglucose (FDG) uptake was poor in the mass of the pulmonary artery, and no other hypermetabolic lesions were noted elsewhere. Our presumptive diagnosis was a massive mural thrombus and a concomitant chronic thromboembolism. Intravenous heparin and recombinant human tissue-type plasminogen activator was subsequently administered. However, the patient needed an emergency operation for sudden aggravation of the vital signs, and the tissue diagnosis was intimal sarcoma with poor clinical outcomes.

## Background

Intimal sarcoma is a rare mesenchymal tumor, and it can arise from the great arteries. The incidence of pulmonary artery intimal sarcoma is almost twice that of the aorta [1]. It usually arises from the intimal layer of the right, left, and main pulmonary arteries. The usual features of these tumors are endoluminal growth and later vessel obstruction or seeding of distal emboli. In addition, in rare cases, it may extend in a retrograde fashion to the pulmonary valve and the right ventricle [1,2].

This tumor is highly malignant and the prognosis is very poor. Survival is usually 12 to 18 months after the onset of the symptoms [1]. This disease mainly occurs in adults with female sex predominance, with the mean age at diagnosis being 48 years [3]. The diagnosis is often delayed and difficult, due to gradually developing symptoms of the pulmonary artery obstruction and right heart failure with a long asymptomatic course.

## Case presentation

A 58-year-old man was admitted with a month’s history of exertional dyspnea, which had been worsening for twenty days. The patient was being medicated for diabetes mellitus and hypertension. He was relatively stable, at New York Heart Association functional class II-III. On physical examination, the patient exhibited tachypnea, with 20 breaths per minute and his oxygen saturation was 92%, his pulse was regular at 95 beats per minute, and his blood pressure was 130/80 mmHg. Auscultation revealed a prominent second heart sound and grade 3/6 systolic murmur over the pulmonary area. A blood count revealed platelets at 57,000/mm^3^, but the rest of the work-up revealed no hematologic evidence of intrinsic hypercoagulability. The N-terminal pro-B-type natriuretic peptide level was 395 pg/ml (normal range: 0–125) and D-dimer was 0.816 μg/ml (normal range: 0–0.5). The chest radiograph revealed atherosclerotic cardiovascular change and mild cardiomegaly. Electrocardiography showed a sinus rhythm.

**Figure 1:**
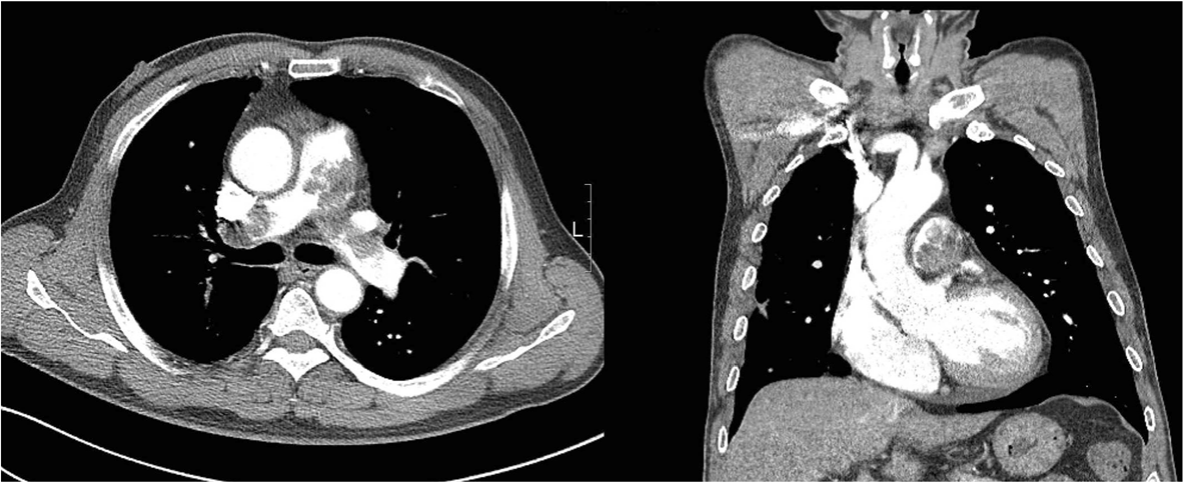
**Chest computed tomography reveals multiple large masses in the main pulmonary artery and both branches.** Main pulmonary artery and left pulmonary artery were almost completely blocked, and the right pulmonary trunk was completely blocked.

Evaluation with transthoracic echocardiography demonstrated a left ventricular ejection fraction of 56%. The right ventricle was enlarged and severely hypokinetic with a mobile echogenic mass in the supravalvular area of the main pulmonary artery. The color Doppler revealed a moderate tricuspid regurgitation. The systolic pulmonary arterial pressure was estimated with 70 mmHg. Subsequent evaluation with contrast-enhanced computed tomography (CT) of the chest demonstrated a large filling defect within the main pulmonary artery, which extended to near total occlusion of the left and right pulmonary artery, separately (Figure 1). There had been no intraluminal mass of the pulmonary artery on a chest CT 8 years earlier. Cardiac magnetic resonance imaging confirmed the mass beginning near the pulmonic valve, centered in the main pulmonary artery and extending into the right ventricular outflow tract, and onto both pulmonary arteries. A bilateral lower-extremity venous duplex scan was negative for acute deep venous thrombosis. Lung scintigraphy showed no visualization of the right lung on a technetium-99 m (99mTc-MAA) perfusion scan, but a ventilation scan showed no evidence of a ventilation defect, which was consistent with ventilation perfusion mismatch.

**Figure 2:**
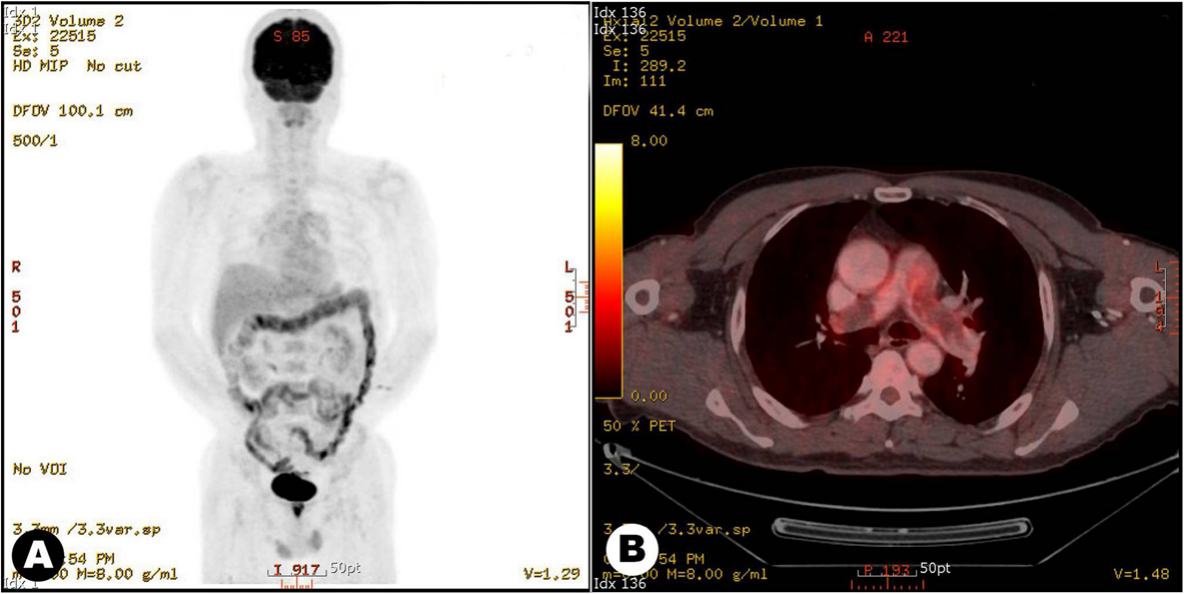
**Maximum intensity projection image of **^**18**^**fluorodeoxyglucose positron emission tomography (FDG-PET) shows no specific uptake of FDG in a chest image (left).** An axial section shows large intraluminal masses in the pulmonary artery without significant FDG-uptake.

For the differentiation of the pulmonary artery mass between the thrombus and neoplasm, ^18^F-fluorodeoxyglucose uptake in positron emission computed tomography (FDG-PET) was performed to confirm the neoplastic features and the distant metastatic extent of the disease. The results of the FDG-PET showed a large mass, but no definite uptake in the mass, and no other hypermetabolic lesions were noted elsewhere (Figure 2). Our presumptive diagnosis was a massive mural thrombus and concomitant chronic thromboembolic disease. Intravenous heparin and recombinant human tissue-type plasminogen activator were subsequently administered.

**Figure 3:**
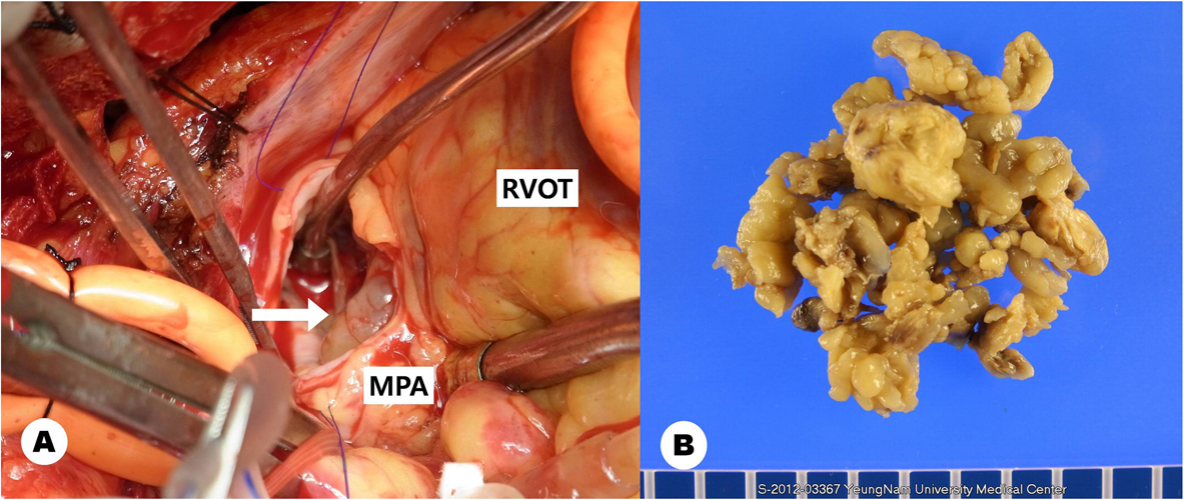
**The large multiple lobulated mass is seen through the main pulmonary artery (arrow).** The mass extended distally to both pulmonary artery branches with a retrograde extension to the pulmonary valve and right ventricular outflow tract (left). Resected tumor masses (right). RV: right ventricle, MPA: main pulmonary artery.

After one day, the clinical presentation of the patient with progressive dyspnea and signs of congestive heart failure led us to decide on an emergent surgical approach. The patient was placed under general anesthesia. A standard median sternotomy and cardiopulmonary bypass was performed with aortic arterial and bicaval venous cannulation. After the aorta was clamped, cold blood cardioplegia was infused. The outer surface of the pulmonary arteries revealed no pathologic features, and longitudinal pulmonary arteriotomy was performed. In the lumen of the main pulmonary artery, a multilobulated whitish-yellow mass (Figure 3) was seen, and most of it involved the vascular inner wall. The main pulmonary artery was almost completely occluded by the mass and the mass had partially adhered to the pulmonic valve annulus. The small polypoid mass presented on the right ventricular outflow tract, and the main mass extended distally to both main pulmonary arteries. The main mass was resected along with part of the pulmonary artery wall but the pulmonary valve annulus was treated by curettage only and the leaflets were preserved. The small mass on the right ventricular outflow tract was resected through the pulmonary valve. The remaining masses on both branched pulmonary arteries were also resected. The defect in the pulmonary artery was repaired with a bovine pericardium patch. Even though as much as possible of both distal pulmonary artery masses were removed, there was no backflow from the right pulmonary artery and scanty backflow from the left pulmonary artery.

The intraoperative frozen biopsy was suggestive of sarcoma. We tried weaning the patient from cardiopulmonary bypass, but had to use extracorporeal membrane oxygenation (ECMO) due to the unstable vital status.

**Figure 4:**
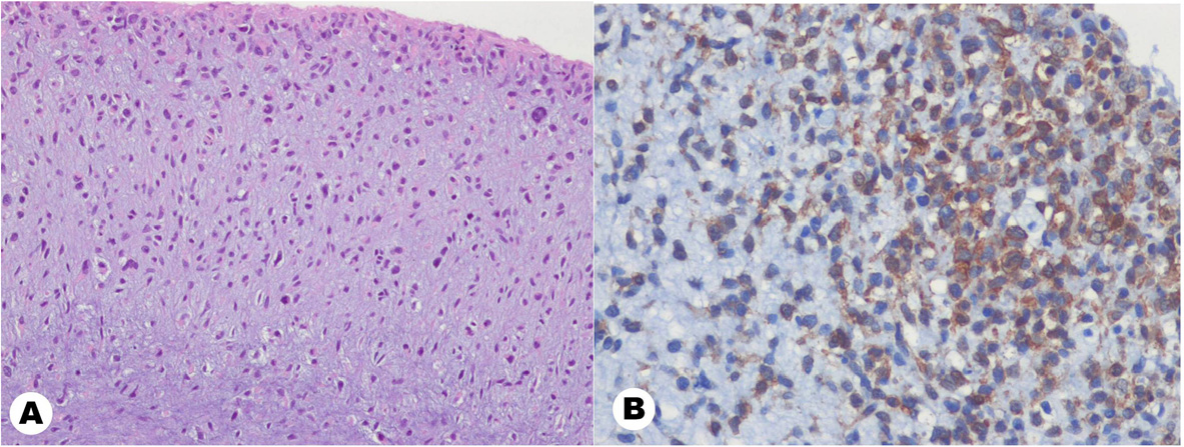
**Histological examination revealed atypical spindle cells with hyperchromatic, pleomorphic nuclei and some mitoses but poor cellularity with marked myxoid tissue.** (hematoxylin-eosin stain; original magnification 100x) (left). Immunostains with CD34 show multifocal positivity for smooth muscle actin as well as focal positivity for desmin consistent with myofibroblastic differentiation (original magnification 200x) (right). These findings are consistent with intimal sarcoma.

Permanent histological findings revealed a non-distinctive myxoid spindle cell sarcoma with a rather lobulated, exophytic growth pattern. Some mitotic activity was seen and pleomorphic tumor cells surrounded the endothelium, in particular (Figure 4). It was also highly malignant, but its cellularity was decreased with marked interstitial myxoid tissue. Immunostains showed multifocal positivity for smooth muscle actin, as well as focal positivity for desmin, consistent with myofibroblastic differentiation, while CD34 and S-100 protein were negative.

Given the poor prognosis and inability to wean the patient from the ECMO, we decided to withdraw the life support system after 5 days.

## Discussions

The clinical features of pulmonary intimal sarcoma are similar, and it is frequently misdiagnosed as chronic pulmonary thromboembolism. It may include right heart failure due to pulmonary hypertension, along with such as a cough, dyspnea, chest pain, hemoptysis, and syncope. However, if concomitant symptoms include fever, cachexia, and anemia but no risk factors for venous thrombosis, malignancy should be considered. In our case, our initial diagnosis was pulmonary thromboembolism due to laboratory tests and radiologic features. There was no hematologic evidence of intrinsic hypercoagulability, but D-dimer was 0.816 ug/ml (normal range: 0–0.5).

Advances in imaging technology, such as echocardiography, contrast-enhanced computed tomography, and magnetic resonance imaging, have supported better diagnoses of masses in the pulmonary artery, but without always differentiating successfully between a thrombus and a malignancy. The FDG-PET is a more reliable method for the confirmation of the neoplastic features and the distant metastatic extent of the disease, due to increased FDG uptake in its malignancy. Even though, FDG is not a tumor-specific agent, FDG-PET is increasingly used. Several authors have reported that FDG-PET has been useful in a diagnostic workup for differentiating among or staging malignant diseases, and monitoring the response to treatment of pulmonary artery intimal sarcoma [4,5]. Among unusual findings, Mathias et. al. [6] reported a pulmonary intimal sarcoma with a lack of uptake of FDG in PET. It also revealed highly malignant cells, but its cellularity was low with marked interstitial myxoid tissue. Also in our patient, the FDG uptake was not significant, and we decided that the mass was a thrombus with distal embolic progression. Even though the character of the tumor was highly malignant, if the cellularity was low and myxoid tissue was marked, a negative FDG-PET study would have been possible. The patient mass did not take up FDG significantly in the FDG-PET, and thus we misdiagnosed it as a thrombus. This mistaken diagnosis could have led to inappropriate therapy, such as anticoagulation and thrombolysis. Unfortunately, there were few treatment options and there are no specific guidelines on the management of pulmonary artery sarcomas. The usual therapy is a surgical approach with adjuvant chemotherapy [7,8]. The prognosis, which depends on the location and vascular extension, is very poor without surgery. The surgical procedures, such as pneumonectomy, palliative stenting, and endarterectomy of the pulmonary artery, depend on the tumor site. Even if patients present with unresectable lesions, debulking surgery might still be needed for hemodynamic improvement. Some cases that have undergone surgery have had a long-term survival of approximately 3 years for isolated local tumors [9]. However, in our patient, both distal pulmonary arteries were completely or partially obstructed with the thrombi. Thus, complete removal of the tumor embolus was impossible, postoperative hemodynamic status was very poor, and ECMO was needed.

## Conclusion

Intimal sarcoma of the pulmonary artery is a rare malignant tumor that may be misdiagnosed as pulmonary thromboembolism, even if advanced imaging technology is used. Even though the FDG is poorly taken up in PET, the possibility of malignancy should be considered. We report a case of pulmonary artery intimal sarcoma that was not initially diagnosed as a malignancy due to poor uptake of FDG in a PET study.

## Consent

Written informed consent was obtained from the patient for publication of this case report and accompanying images. A copy of the written consent is available for review by the Editor-in-Chief of this journal.

## Competing interests

The authors declare that they have no competing interests.

## Authors’ contributions

DL, DS and SP wrote the draft of the manuscript and obtained the written consent. TJ, JL and JC performed the literature review and participated in the manuscript writing and helped to the final writing of the paper and gave final approval of the manuscript. All authors have read and approved the final manuscript.
